# Risk factors for fatty pancreas and effects of fatty infiltration on pancreatic cancer

**DOI:** 10.3389/fphys.2023.1243983

**Published:** 2023-07-17

**Authors:** Nao Otsuka, Kyoko Shimizu, Makiko Taniai, Katsutoshi Tokushige

**Affiliations:** ^1^ Department of Internal Medicine, Institute of Gastroenterology, Tokyo Women’s Medical University, Tokyo, Japan; ^2^ Shinjuku Mitsui Building Clinic, Tokyo, Japan

**Keywords:** pancreatic steatosis, pancreatic cancer, macrophage, pancreatic acinar cell, autophagy dysfunction

## Abstract

**Objective:** This study clarified the risk factors and pathophysiology of pancreatic cancer by examining the factors associated with fatty pancreas.

**Methods:** The degree of fatty pancreas, background factors, and incidence of pancreatic cancer were examined among nonalcoholic fatty liver disease (NAFLD) patients (n = 281) and intraductal papillary mucinous neoplasm (IPMN) patients with a family history of pancreatic cancer (n = 38). The presence of fatty pancreas was confirmed by the pancreatic CT value/splenic CT value ratio (P/S ratio). Immunohistochemical staining was performed on 10 cases with fatty pancreas, confirmed via postoperative pathology.

**Results:** Fatty pancreas occurred in 126 patients (44.8%) in the NAFLD group who were older (*p* = 0.0002) and more likely to have hypertension (*p* < 0.0001). The IPMN group had 18 patients (47.4%) with fatty pancreas, included more men than women (*p* = 0.0056), and was more likely to have patients with hypertension (*p* = 0.0010). On histological examination, a significant infiltration of adipocytes into the acini from the pancreatic interstitium induced atrophy of the pancreatic parenchyma, and both M1 and M2 macrophages were detected in the area where adipocytes invaded the pancreatic parenchyma. Accumulation of p62 and increased positive staining of NQO1 molecules related to autophagy dysfunction were detected in pancreatic acinar cells in the fatty area, acinar-ductal metaplasia, and pancreatic cancer cells. The rate of p62-positive cell area and that of NQO1-positive cell area were significantly higher in the fatty pancreatic region than those in the control lesion (pancreatic region with few adipocyte infiltration). Furthermore, the rate of p62-positive cell area or that of NQO1-positive cell area showed strong positive correlations with the rate of fatty pancreatic lesion. These results suggest that adipocyte invasion into the pancreatic parenthyme induced macrophage infiltration and autophagy substrate p62 accumulation. High levels of NQO1 expression in the fatty area may be dependent on p62 accumulation.

**Conclusion:** Hypertension was a significant risk factor for fatty pancreas in patients with NAFLD and IPMN. In fatty pancreas, fatty infiltration into the pancreatic parenchyme might induce autophagy dysfunction, resulting in activation of antioxidant proteins NQO1. Thus, patients with fatty pancreas require careful follow-up.

## 1 Introduction

Fatty accumulation in the pancreas, named fatty pancreas, is caused by various conditions including alcohol abuse, infections, pancreatitis, congenital diseases, medicines, and metabolic diseases (Dite et al., 2020). Nonalcoholic fatty pancreas disease (NAFPD) has recently attracted attention in relation to obesity, diabetes, and metabolic syndrome (van Geenen et al., 2010; Dite et al., 2020). With the advancement of imaging tests, such as echocardiography, CT, and MRI, fatty pancreas has become relatively easy to diagnose. A decrease in the pancreatic CT value on plain abdominal CT images correlates with the degree of pancreatic fatty infiltration (Kim et al., 2014; Mori et al., 2019). In histological evaluation of fatty pancreas, when the ratio of pancreatic CT value/splenic CT value is less than 0.8, moderate or more fatty deposits are observed in the pancreatic parenchyma (Mori et al., 2019).

Fatty pancreas is observed in 16%–35% of Asians (Wang et al., 2014; Lesmana et al., 2015; Zhou et al., 2016), while 50%–80% of patients with non-alcoholic fatty liver disease (NAFLD) are reported to have fatty pancreas comorbidity (Al-Haddad et al., 2009; Uygun et al., 2015). Obesity, diabetes, and metabolic syndrome have been suggested as causes of fatty pancreas, but the etiology and pathology remain unclear. Meanwhile, according to some reports (Hori et al., 2014; Takahashi et al., 2018), fatty pancreas may be a risk factor for pancreatic cancer, thereby drawing attention to the clinical significance and importance of fatty pancreas.

Thus, we aimed to clarify the risk and pathophysiology of pancreatic cancer by examining the frequency and associated factors of fatty pancreas in non-pancreatic and pancreatic diseases. In a non-pancreatic disease group, we investigated the frequency and background factors of fatty pancreas in NAFLD and the incidence of pancreatic cancer. Risk factors for pancreatic cancer include family history of pancreatic cancer, intraductal papillary mucinous neoplasm (IPMN), obesity, etc. In relation to fatty pancreas, patients with IPMN are frequently associated with fatty pancreas (Kashiwagi et al., 2018). Therefore, we examined the frequency of fatty pancreas and cancer risk in IPMN patients with a family history of pancreatic cancer. In addition, we conducted an immunohistological examination using pancreatic resection specimens for histological pathology of fatty pancreas.

## 2 Materials and methods

### 2.1 Study population

We analyzed the data of 281 patients diagnosed with fatty liver by liver biopsy between May 2007 and September 2010, as well as those of 38 patients with IPMN having a family history of pancreatic cancer in their first- or second-degree relatives between January 2016 and December 2019. The diagnosis of IPMN was made on the basis of CT, magnetic resonance cholangiopancreatography, and endoscopic ultrasonography findings, according to the criteria in the international consensus guidelines 2017 (Tanaka et al., 2017).

In histological examination, from 2011 to 2020, immunohistochemical staining was performed on 10 cases in which fatty pancreas was confirmed via postoperative pathology among the cases that underwent pancreatic resection for pancreatic disease. For the comparison between fatty and normal pancreas in the non-neoplastic lesion, we used a region distant from the main lesion targeted for pancreatic resection and without pancreatic duct lesions.

### 2.2 Clinical data collection

The use of clinical data and pancreatic tissue blocks after pancreatectomy for analysis in our study was approved by the Ethics Review Committee of the Tokyo Women’s Medical University. The materials are from the patients who had given general consent for the research use of their leftover samples. All clinical investigations were conducted in accordance with the principles of the Declaration of Helsinki.

The clinical data of the participants were extracted from electronic medical records: age, sex, body mass index (BMI) [kg/m2], clinical diagnosis of pancreatic diseases, or NAFLD. The comorbidities we examined were type 2 diabetes mellitus, hypertension, and dyslipidemia with treatment history.

### 2.3 CT alue measurement method and definition of fatty pancreas

**FIGURE 1:**
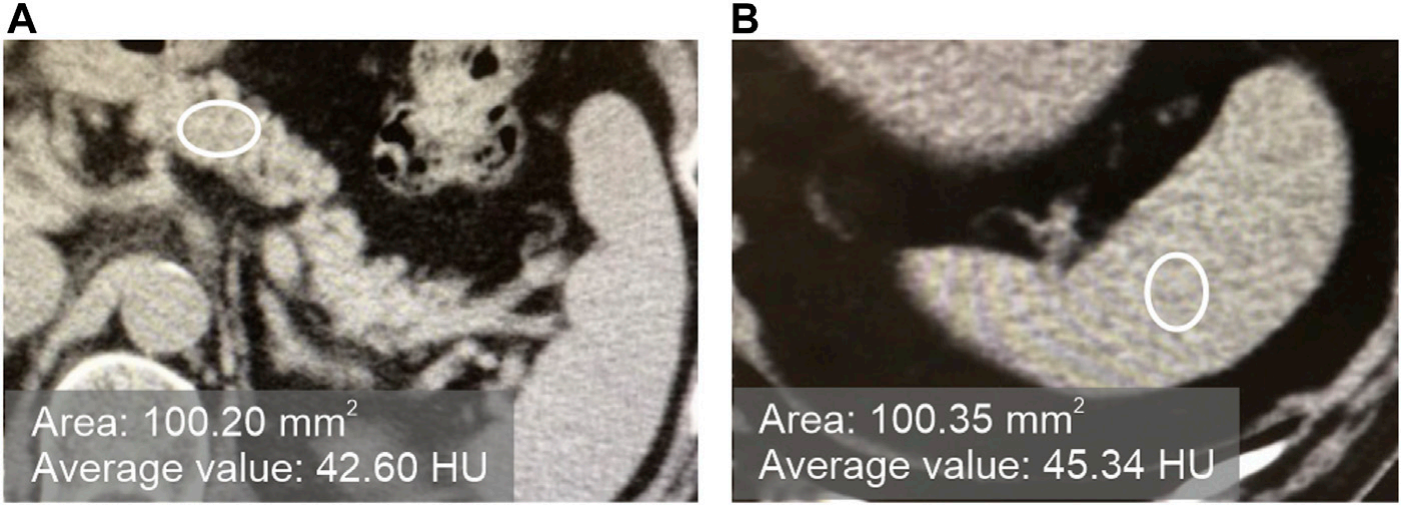
Measurement of the CT values of the pancreas and spleen in plain CT. [**(A)**: pancreas, **(B)** spleen].

In plain CT images at the first visit, pancreatic CT value/splenic CT value ratio (hereinafter referred to as P/S ratio) was defined as < 0.8 for the fatty pancreas group (F group) and ≥0.8 for the non-fatty pancreas group (N group). As shown in Figure 1, the CT value was measured by setting three regions of interest (ROI) on each of the pancreas and spleen on plain CT images, and ROI of approximately 100 mm^2^ on regions that did not overlap with vessels, cystic lesions, or neoplastic lesions. The CT value within the ROI was measured, and the average value of the three points was calculated as the “pancreatic CT value” and the “spleen CT value.” The thickness (mm) of the pancreatic body measured by CT was used as an index of pancreatic volume.

### 2.4 Histological examination

Pancreatic specimens were fixed in 4% paraformaldehyde (PFA) and embedded in paraffin, and then the sections were deparaffinized and rehydrated through xylene and ethanol. After staining with hematoxylin-eosin, the samples were evaluated histologically. Immunohistochemical staining was performed with the Histofine®Simple Stain MAX-PO (MULTI) (NICHIREI BIOSCIENCES, Japan). We used the antigen retrieval step recommended by the antibody datasheet for each staining. The sections were incubated with mouse monoclonal anti-CD11c antibody (NCL-L-CD11c-563, Leica Biosystems), mouse monoclonal anti-CD163 (NCL-L-CD163, Leica Biosystems), mouse monoclonal anti-sequestosome 1(SQSTM1/p62) antibody (sc-28359, Santa Cruz Biotechnology, Inc.), rabbit polyclonal anti-WD repeat and FYVE domain-containing protein 3 (WDFY3) antibody (Novus Biologicals), rabbit monoclonal anti-cleaved caspase-3 antibody (Cell Signaling Technology, Inc.), rabbit polyclonal anti-apoptosis-inducing factor (AIF) antibody (#4642, Cell Signaling Technology, Inc.), rabbit polyclonal anti-microtubule-associated protein1 light chain 3 (LC3) antibody (PM036, Medical & Biological Laboratories), rabbit polyclonal anti-NAD(P)H dehydrogenase quinone 1 (NQO1) antibody (11451- 1- AP, Proteintech), or anti-perilipin-1 antibody (#9349, Cell Signaling Technology, Inc.). After washing with phosphate-buffered saline, the sections were incubated with a horseradish-peroxidase-labeled biotinylated secondary antibody. The color reaction was developed in 3,3′-diaminobenzidine (DAB), and the sections were counterstained with hematoxylin. Normal animal immunoglobulin was substituted for the primary antibodies to establish a negative control. We performed TdT-mediated dUTP nick end labeling (TUNEL) assay using the *In Situ* Apoptosis Detection Kit (#MK500, TAKARA BIO, Japan) for detection of apoptosis.

### 2.5 Pathological image analysis

**FIGURE 2:**
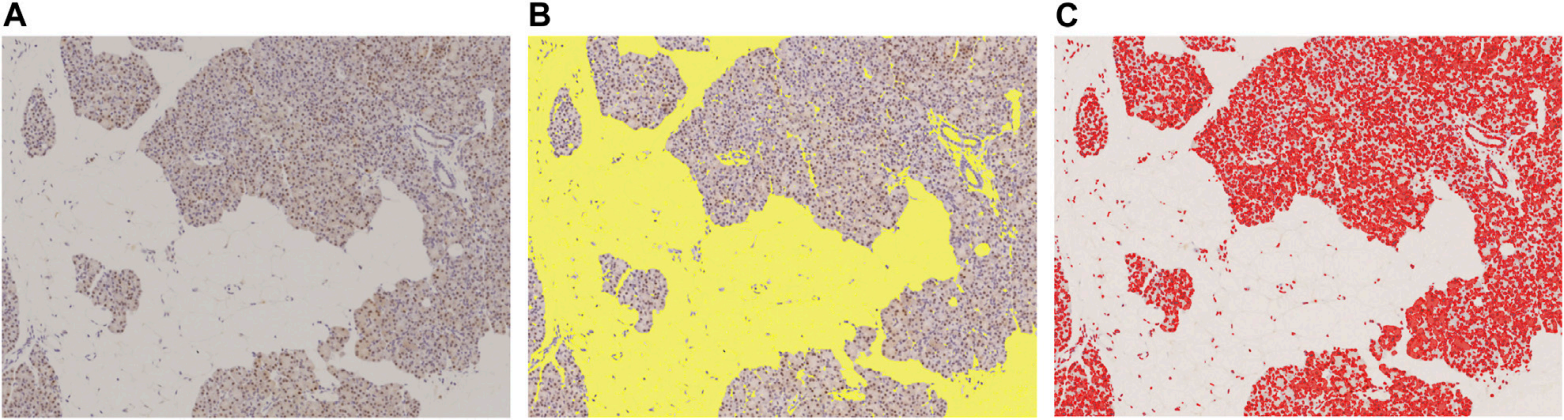
Measured with image analysis software to calculate area fraction. **(A)**: Original DAB-immunostained slide, **(B)**: Slide scanned with color scanner shows total area of fat of pancreatic parenchyma and stroma (yellow), **(C)**: DAB-positive stained area (red). This slide was stained with p62. These areas were measured with image analysis software to calculate area fraction. In this slide, the blank area (fat and stroma) was 43.6%, and the p62 positive area, 16.8%.

Using NIS-Elements (Nikon), we binarized the slide images with a threshold set in RBG color, and then calculated the areas of the extracted objects. The binarization threshold was preserved, and all slides were extracted under same conditions. The area of stroma and fat other than the pancreatic parenchyma was defined as the blank area, while DAB substrate-stained portions were treated as the positive area (Figure 2).

### 2.6 Statistical method

We conducted a chi-squared test for the nominal variables and the presence or absence of fatty pancreas in the patient background factors, and univariate analysis using the *t*-test for continuous variables and presence or absence of fatty pancreas. We extracted independent factors using the stepwise method from the variables identified as significant factors by univariate analysis, and then used logistic regression analysis for those independent factors and the presence or absence of fatty pancreas. In both univariate and multivariate analyses, a *p*-value of less than 0.05 was defined as significant.

In the immunohistological examination, we calculated the correlation coefficient (r) between the staining positive area % and blank area %. We observed no correlation when the absolute value was <0.3. Weak correlation was defined as ≥ 0.3 and <0.5, significant correlation as ≥ 0.5 and <0.7, and strong correlation as 0.7 or more. We used JMP Pro version 15.0.0 as statistical software.

## 3 Results

### 3.1 Risk factors for fatty pancreas in patients with NAFLD

Table 1 shows the background of the 281 cases of NAFLD. Table 2 shows the univariate analysis results for patients with fatty pancreas (group F) and those without (group N). No significant sex difference was observed between the two groups. As shown in Table 2, the average age was higher in the F group than in the N group (*p* = 0.0002), while the thickness of the pancreatic body was thinner in the F group (*p* = 0.0025). With respect to comorbidities, hypertension was at a higher frequency in the F group (OR 3.01, 95% CI: 1.84–4.93, *p* < 0.0001). However, there were no significant differences in BMI, diabetes, and dyslipidemia between both groups. In the multivariate analysis, only hypertension was extracted as a significant independent factor (*p* = 0.0005).

**TABLE 1:** Characteristics of the NAFLD patients (n = 281).

	Mean ± SD	n	%
Male		152	53.8
Age (years)	53.9 ± 15.5		
BMI	27.0 ± 5.1		
P/S ratio	0.73 ± 0.31		
P/S ratio <0.8		126	44.8
Thickness of P-body (mm)	18.2 ± 4.5		
Type 2 diabetes mellitus		150	53.4
Hypertension		153	54.4
Dyslipidemia		193	68.7

Note: NAFLD, non-alcoholic fatty liver disease; SD, standard deviation; BMI, body mass index, P/S = pancreatic CT, value/splenic CT, value, P-body = pancreatic body.

**TABLE 2:** Association among risk factors and presence of fatty pancreas in NAFLD patients (n = 281).

	Fatty pancreas			
	+	−		
	n = 126	n = 55	Odds (95% CI)	*p*-Value
Male (n = 152)	71 (56.3%)	81 (52.3%)	1.18 (0.53–1.36)	0.4937
Age (years), Mean ± SD	58.0 ± 13.9	51.1 ± 16.1		0.0002
BMI, Mean ± SD	27.6 ± 4.9	26.6 ± 5.0		0.1035
Thickness of P-body (mm), Mean ± SD	17.3 ± 4.3	18.9 ± 4.6		0.0025
Type 2 diabetes mellitus (n = 150)	74 (58.7%)	76 (49.0%)	1.48 (0.92–2.38)	0.1051
Hypertension (n = 153)	87 (69.0%)	66 (42.6%)	3.01 (1.84–4.93)	<0.0001
Dyslipidemia (n = 193)	91 (72.2%)	102 (65.8%)	1.35 (0.81–2.25)	0.2488

Note: NAFLD, non-alcoholic fatty liver disease; CI, confidence interval; BMI, body mass index; SD, standard deviation, P-body = pancreatic body.

### 3.2 Risk factors for fatty pancreas in IPMN patients with a family history of pancreatic cancer

Table 3 shows the background of 38 IPMN patients. Almost half (18, 47.4%) of them had fatty pancreas. Table 4 shows the results of the univariate analysis. The F group was predominantly male (OR 8.00, 95% CI: 1.84–34.79, *p* = 0.0056) and showed no significant difference in age. The thickness of the pancreatic body was thinner in the F group (*p* = 0.0017). Regarding comorbidities, hypertension was more common in the F group (OR 14.73, 95% CI: 2.97–73.21, *p* = 0.0010), with no significant difference in BMI and the presence or absence of diabetes, dyslipidemia, and NAFLD. In the multivariate analysis, the thickness of the pancreatic body (*p* = 0.0036) and hypertension (*p* = 0.0007) were also extracted as significant independent factors.

**TABLE 3:** Characteristics of IPMN patients with family history of pancreatic cancer (n = 38).

	Mean ± SD	n	%
Male		16	42.1
Age (years)	64.1 ± 12.1		
BMI	21.8 ± 3.1		
P/S ratio	0.67 ± 0.38		
P/S ratio <0.8		18	47.4
Thickness of P-body (mm)	15.6 ± 3.8		
Type 2 diabetes mellitus		11	28.9
Hypertension		16	42.1
Dyslipidemia		10	26.3
NAFLD		4	10.5

Note: IPMN, intraductal papillary mucinous neoplasm; SD, standard deviation; BMI, body mass index, P/S = pancreatic CT, value/splenic CT, value; NAFLD, non-alcoholic fatty liver disease, P-body = pancreatic body.

**TABLE 4:** Association among risk factors and the presence of fatty pancreas in IPMN patients with family history of pancreatic cancer (n = 38).

	Fatty pancreas			
	+	−		
	n = 18	n = 20	Odds (95% CI)	*p*-Value
Male (n = 16)	12 (66.7%)	4 (20.0%)	8.00 (1.84–34.79)	0.0056
Age, Mean ± SD	67.2 ± 10.1	61.3 ± 13.2		0.1365
BMI, Mean ± SD	21.2 ± 2.0	22.5 ± 4.0		0.3494
Thickness of P-body (mm), Mean ± SD	13.7 ± 3.2	17.4 ± 3.6		0.0017
Type 2 diabetes mellitus (n = 11)	7 (38.9%)	4 (20.0%)	2.55 (0.60–10.84)	0.2062
Hypertension (n = 16)	13 (72.2%)	3 (15.0%)	14.73 (2.97–73.21)	0.0010
Dyslipidemia (n = 10)	7 (38.9%)	3 (15.0%)	3.61 (0.76–17.00)	0.1050
NAFLD (n = 4)	2 (11.1%)	2 (10.0%)	1.13 (0.14–8.94)	0.9113

Note: IPMN, intraductal papillary mucinous neoplasm; CI, confidence interval; SD, standard deviation; BMI, body mass index, P-body = pancreatic body, NAFLD, non-alcoholic fatty liver disease.

### 3.3 Incidence of pancreatic cancer in patients with fatty pancreas

Of the 281 NAFLD cases, three developed pancreatic cancer, all of whom had fatty pancreas. Pancreatic cancer occurred in three of the 126 patients with fatty pancreas, whereas pancreatic cancer did not occur in the 155 patients without fatty pancreas. Furthermore, four of the 38 IPMN patients with a family history of pancreatic cancer had endoscopic retrograde cholangiopancreatography for pancreatic juice cytology after more than 6 months from the first visit. High-grade dysplasia was detected, and the pathological diagnosis after pancreatectomy was noninvasive intraductal papillary mucinous carcinoma in all four cases. High-grade dysplasia occurred in three cases (16.7%) from 18 cases with fatty pancreas and one case (5%) from 20 cases with non-fatty pancreas, showing that pancreatic cancer occurred at a high proportion arising from the IPMN of fatty pancreas. However, there was no statistical difference in the occurrence of pancreatic cancer in fatty pancreas (Table 5).

**TABLE 5:** Occurrence of malignancy in IPMN patients with family history of pancreatic cancer (n = 38).

	Malignancy			
	+	-		
	n = 4	n = 34	Odds (95% CI)	*p*-Value
Fatty pancreas (n = 18)	3 (75.0%)	15 (44.1%)	3.80 (0.36–40.33)	0.2680

Note: IPMN, intraductal papillary mucinous neoplasm; CI, confidence interval.

### 3.4 Results of immunohistochemical examination of fatty pancreas

Immunohistological examination was performed in resected pancreas specimens from 10 cases with pancreatic diseases. The 10 cases consisted of three cases with pancreatic cancer, two with chronic pancreatitis, three with intraductal papillary mucinous carcinoma, one with autoimmune pancreatitis, and one with pancreatic intraepithelial neoplasia (PanIN). In all cases, the inflammatory parameters WBC and CRP were within the normal range when the pancreatic resection was performed.

**FIGURE 3:**
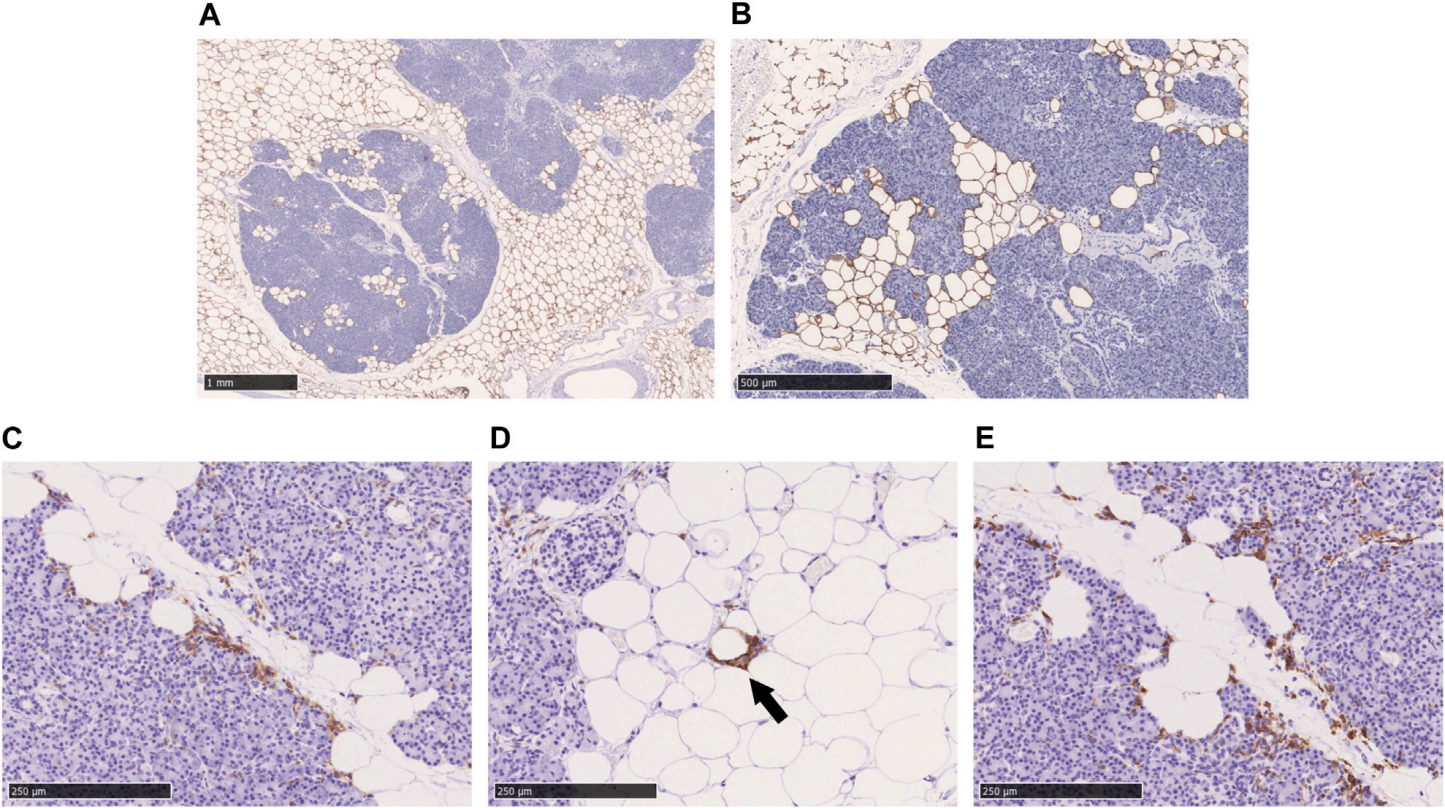
Immunohistochemical staining with perilipin, CD11c, and CD163 in the fatty infiltration of the pancreas. **(A, B)**: Immunohistochemical staining with perilipin, **(C)**: CD11c-staining, **(D)**: CD11c-staining, black arrow shows crown-like structure. **(E)**: CD163-staining. Scale bar: 1 mm in **(A)**, 500 μm in **(B)**, 250 μm in **(C)**, **(D)**, and **(E)**.

Perilipin is located at the periphery of lipid droplets and is highly expressed in adipocytes. Immunohistochemical staining of perilipin showed a significant infiltration of adipocytes into the acini from the pancreatic interstitium, resulting in pancreatic parenchymal atrophy. No lipid droplet deposition within the pancreatic acinar cells was confirmed (Figures 3A,B). As shown in Figures 3C–E, many CD11c-positive M1 macrophages and CD163-positive M2 macrophages were present in abundance at the boundary between adipocytes and acinar cells in the pancreatic parenchyma. Crown-like structures (CLS), composed of macrophages surrounding dead or dying adipocytes, were rarely observed (Figure 3D).

**FIGURE 4:**
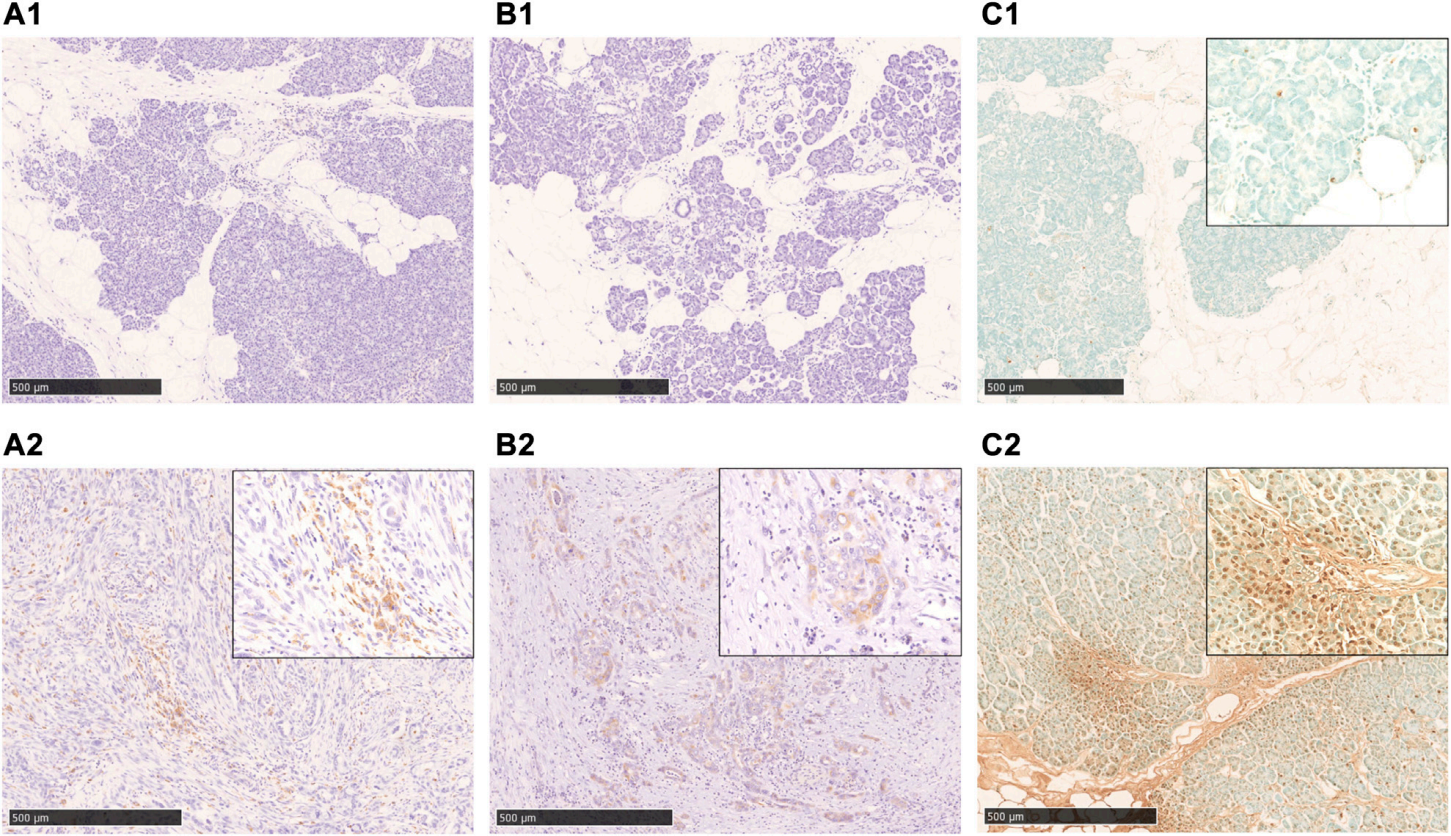
Immunohistochemical staining with cleaved caspase 3, AIF, and TUNEL-staining. **A1**, **A2**: cleaved caspase 3-staining, **B1**, **B2**: AIF-staining, **C1**, **C2**: TUNEL-staining. In the fatty infiltration area of the cases in the non-neoplastic lesion, both cleaved caspase 3(**A1**) and AIF(**B1**)-staining are negative, and also TUNEL-staining is rarely positive (**C1**). In cases of pancreatic cancer **(A2, B2)** and chronic pancreatitis **(C2)**, a significant positive staining in the cancer cells and acinar cells was observed. Scale bar is 500 μm.

**FIGURE 5:**
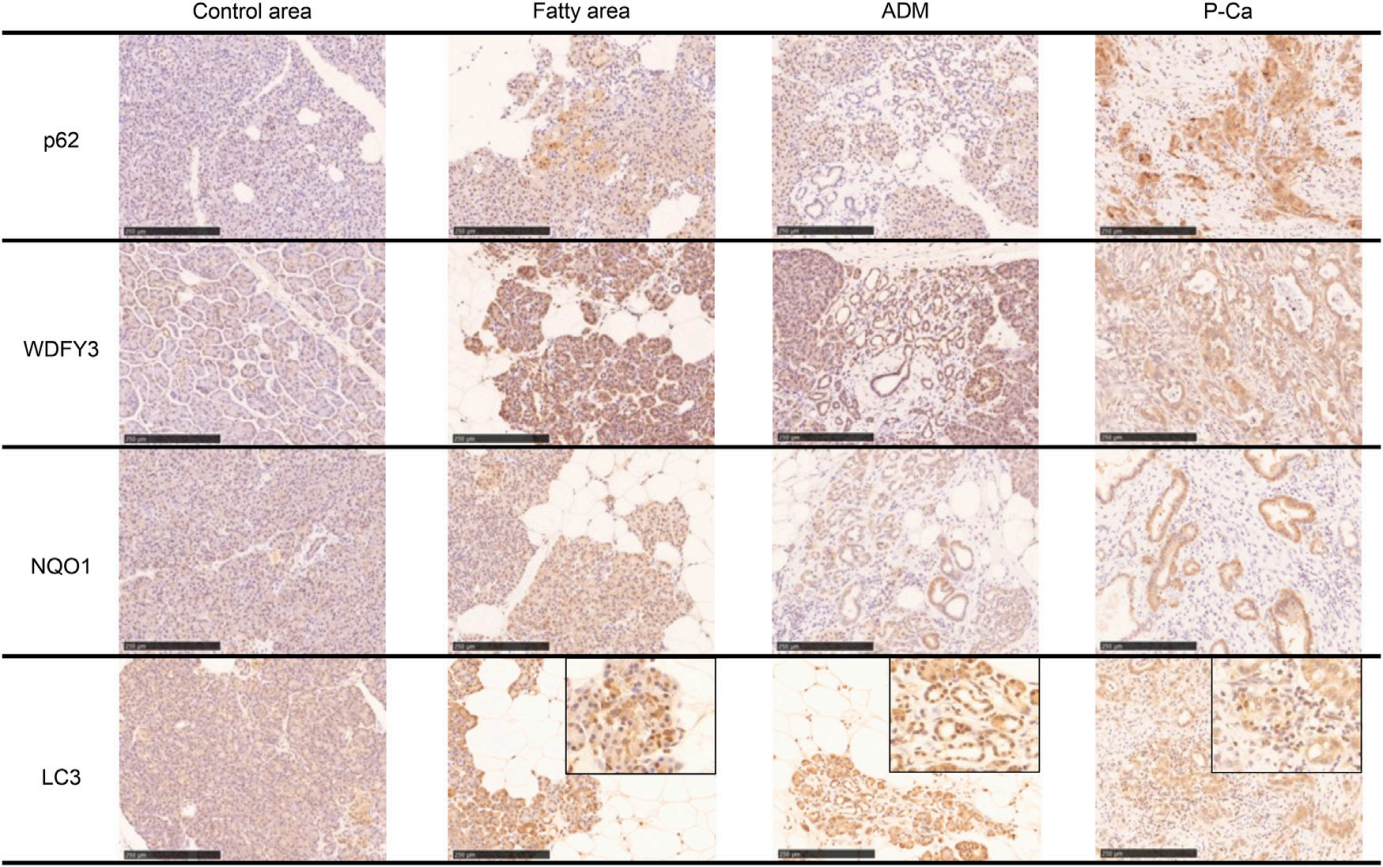
Immunohistochemical staining with p62, WDFY, NQO1, and LC3. These markers are strongly positive in the boundary of the fatty area, acinar-ductal metaplasia, and pancreatic cancer. Scale bar is 250 μm.

Immunostaining of cleaved caspase 3 and AIF, which are central enzymes in the process of apoptosis, was negative in areas with fatty infiltration, and TUNEL-positive cells were few, whereas fibrosis sites and pancreatic cancer showed positive expression (Figure 4). Conversely, autophagy-related molecules, such as p62, WDFY3, and NQO1, were positive in pancreatic acinar cells adjacent to fatty infiltration. Notably, the number of positive cells was small in acinar cells in areas without fatty infiltration. LC3 showed strong staining in the cytoplasm and acinar-ductal metaplasia (ADM) of acinar cells near the boundary with adipocytes infiltration where pancreatic parenchyma tended to be replaced by adipocytes. In contrast, staining was negative in acinar cells in areas without fatty infiltration. We also confirmed a high degree of positivity in pancreatic cancer cells in pancreatic cancer cases. Similar to LC3, we found that p62, WDFY3, and NQO1 were also strongly positive in ADM and pancreatic cancer cells (Figure 5).

**FIGURE 6:**
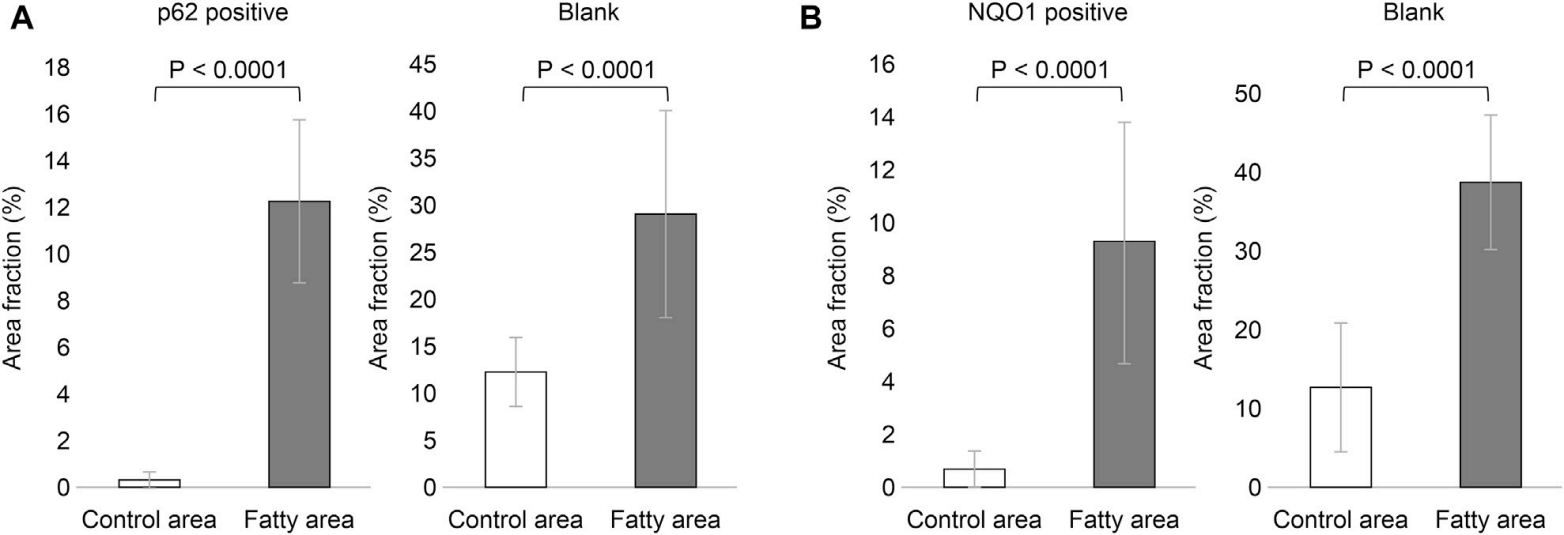
Relation of area fraction of p62 and NQO1 positive with blank in control and fatty pancreas. **(A)**: p62 positive area (%) (mean ± SD): Control area 0.262 ± 0.408 vs. Fatty area 12.317 ± 3.557, *p* < 0.0001. Blank area (%) (mean ± SD): Control area 12.333 ± 3.662 vs. Fatty area 29.238 ± 11.081, *p* < 0.0001. **(B)**: NQO1 positive area (%) (mean ± SD): Control area 0.538 ± 0.745 vs. Fatty area 9.369 ± 4.588, *p* < 0.0001. Blank area (%) (mean ± SD): Control area 13.062 ± 8.352 vs. Fatty area 39.394 ± 8.524, *p* < 0.0001.

**FIGURE 7:**
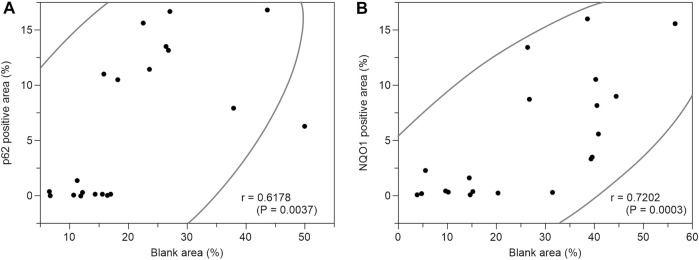
Correlation coefficients between p62 positive area (%) or NQO1 positive area (%) and blank area (%). **(A)**: There is a strong correlation between p62 positive area (%) and blank area (%) (r = 0.6178, *p* = 0.0037). **(B)**: There is a strong correlation between NQO1 positive area (%) and blank area (%) (r = 0.7202, *p* = 0.0003).

Based on the above staining patterns, we examined differences in p62 and NQO1-positive areas between areas with fatty infiltration (fatty area) and areas without fatty infiltration (control area) in 10 cases. The area fraction of p62-positive was significantly higher in the fatty pancreatic region than that in the control area. As for the area fraction of the blank area that means the stroma mainly composed of adipocytes infiltration, it was confirmed that the area fraction of blank area was significantly higher in the fatty region than in control area (Figure 6A). We also observed a strong correlation between the p62-positive and blank areas (r = 0.6178, *p* = 0.0037), as shown in Figure 7A. Similarly, the NQO1-positive and blank area fraction were significantly higher in the fatty pancreas area than in the control area (Figure 6B). We also noted a strong correlation between the NQO1-positive and blank areas (r = 0.7202, *p* = 0.0003) (Figure 7B).

## 4 Discussion

### 4.1 Risk factors for fatty pancreas in patients with NAFLD or IPMN

It is well established that older age and metabolic syndrome, including obesity, diabetes, dyslipidemia, and hypertension, are associated with both NAFLD and NAFPD. In the present study, age and hypertension were significant risk factors of NAFPD in NAFLD patients. Hypertension is also reported as a more important risk factor than diabetes or lipid parameters in NAFPD (Lesmana et al., 2015). Ischemia in hypertension causes numerous changes in various organs, such as the renin-angiotensin system (RAS), a critical regulator of systemic vascular resistance and blood pressure. The RAS is expressed in adipose tissue and the pancreas. Angiotensin 2 regulates adipocyte differentiation, adipokine secretion, and insulin resistance (Pahlavani et al., 2017). Specific angiotensin II receptor binding sites are known to be present in the islet cells, acinar cells, duct cells, pancreatic vasculature, and epithelia of the pancreatic ductal system (Carlsson et al., 1998; Gletsu et al., 2005). As hypertension is a significant risk factor for fatty pancreas among patients with NAFLD or IPMN who have metabolic syndrome, appropriate management is considered necessary.

### 4.2 Fatty pancreas and pancreatic cancer

Various reports have attempted to address the relationship between fatty pancreas and pancreatic diseases, while, in recent years, attention has been paid to the relationship with pancreatic carcinogenesis. Hori et al. (2014) compared cases of cancer and non-cancerous pancreatic resection cases and found that the amount of intrapancreatic fat is higher in resected cases of pancreatic cancer (median pancreatic fat content 25.8% vs. 15.0%, *p* < 0.001). They also showed that the larger the amount of intrapancreatic fat, the higher the pancreatic cancer odds ratio (intrapancreatic fat <10% vs. ≥ 20%: OR 6.1, 95% CI 2.4–15.2, *p* < 0.001).

In relation to pancreatic intraepithelial neoplasia (PanIN), a premalignant lesion, it has been discussed whether lobular atrophy and fat replacement in the surrounding tissue are preceded by pancreatic steatosis or whether PanIN causes pancreatic steatosis. Rebours et al. (2015) reported that pancreatic fatty infiltration is not limited to the area around PanIN, pathologically, suggesting that PanIN originates from fatty pancreas. Pancreatic fatty infiltration occurs interlobularly and intralobularly, and the latter has been shown to be a risk factor for PanIN (OR 17.86, 95% CI: 4.935–88.12, *p* < 0.0001) (Rebours et al., 2015).

IPMN is a risk factor for pancreatic cancer, while fatty pancreas is associated with malignant transformation of IPMN. A comparison between IPMN and non-cystic cases showed that apolipoprotein A1 and HDL-C are low in IPMN cases and correlated with pancreatic fat deposition, suggesting that IPMN induces pancreatic fat deposition (Kashiwagi et al., 2018). In other words, IPMN and fatty pancreas may overlap as risk factors for pancreatic cancer.

In the present study, although not significant, the incidence of pancreatic cancer tended to be high in patients with fatty pancreas. Furthermore, older age was shown to be a significant risk factor of fatty pancreas in patients with NAFLD. The pathological age-related non-neoplastic change in the pancreas is described as fatty replacement, atrophy of acinar parenchyma, ADM, duct ectasia, and metaplasia of exocrine pancreas, as well as changes in islet cells (Matsuda, 2019). ADM is characterized by dilatation of an acinus with formation of a duct-like tubular structure and is at risk of developing into PanIN (Ardito et al., 2012).

Although a strong relationship has been noted between pancreatic fatty infiltration and pancreatic carcinogenesis, no previous studies have revealed the detailed pathophysiology of what kind of carcinogenesis process is affected. Therefore, we focused on the histopathological effects of pancreatic fat infiltration on autophagy and antioxidant mechanisms.

### 4.3 Fatty infiltration and inflammatory macrophage activation in fatty pancreas

Perilipin is an intramembrane protein that surrounds lipid droplets, and studies have identified perilipin1–5. Perilipin1-positive lipid droplets are observed around lipid droplets in hepatocytes in NAFLD (Fujii et al., 2009). However, in our study, perilipin1 staining in pancreatic acinar cells was not observed at any site in fatty pancreas. As such, unlike NAFLD, the main pathophysiology of fatty pancreas is thought to be fat proliferation and infiltration into pancreatic parenchyma, rather than accumulation of lipid droplets in pancreatic acinar cells.

Obesity increases CD11c-positive M1 macrophages and inflammatory cytokine production from adipose tissue, while also promoting insulin resistance (Castoldi et al., 2016). In fatty pancreas, we observed many CD11c-positive M1 macrophages around the adipose tissue infiltrating the pancreatic parenchyma, whereas CLSs are rarely observed in fatty pancreas. CLSs comprise a characteristic finding of NAFLD, in which hepatocytes containing large lipid droplets undergo cell death and M1 macrophages surround the dead hepatocytes (Itoh et al., 2013). However, given the exceedingly low amount of lipid droplets in the acinar cells of fatty pancreas found in the present study, the M1 macrophages that phagocytized the acinar cells that had undergone cell death would not exhibit the morphology of CLS. M2 macrophages secrete pro-fibrogenic factors, such as TGF-β, CCL17, and CCL22, and they may be involved in the progression of fatty pancreatic fibrosis (Mantovani et al., 2004; Mosser and Edward, 2008). Although in the present study, we observed pancreatic fibrosis in chronic pancreatitis and some areas around pancreatic cancer cells, no fibrosis was observed in areas with just fatty infiltration in the examined tissue specimens.

### 4.4 Autophagy failure and increased NQO1 expression in fatty pancreas

Autophagy helps supply the energy and amino acids necessary for survival by increasing induction during starvation. Since p62 can bind to the autophagosome membrane-bound protein LC3, a ubiquitin chain, it is a selective autophagic adapter molecule that degrades ubiquitinated cargoes. Impaired autophagy induces gene expression of antioxidant proteins involved in defense, accompanied by abnormal accumulation and aggregation of proteins. A remarkable amount of p62 accumulates in tissues with autophagy dysfunction, while previous studies have clarified a correlation between p62-mediated selective autophagy and the oxidative stress response system Keap1–Nrf2 pathway (Jain et al., 2010; Komatsu et al., 2010; Lau et al., 2010; Taguchi et al., 2012; Bae et al., 2013; Ichimura et al., 2013). This interaction inhibits the degradation of transcription factors that promote gene expression of antioxidant proteins, and excessive accumulation of p62 in cells indirectly induces gene expression of antioxidant proteins. This stress defense system was revealed by Komatsu et al. (2010). These phenomena are known as pathological characteristics of malignant tumors. Nrf2 enhances gene expression of xenobiotic-metabolizing enzymes—such as glutathione-S-transferase (a detoxification enzyme for electrophiles), NQO1, glutathione synthetase, and multidrug resistance-associated protein 1—and detoxifies electrophiles (Itoh et al., 2010).

In NQO1-expressing cells, increased reactive oxygen species cause single-stranded DNA damage (Silvers et al., 2017), while NQO1 is expressed at increased levels in many cancers, including pancreatic cancer. In pancreatic cancer, it is highly expressed in pancreatic cancer cells and PanIN, and NQO1 inhibitors suppress the progression of pancreatic cancer. Indeed, NQO1 expression is an independent factor for poor pancreatic cancer prognosis (Cullen et al., 2003; Lewis et al., 2004; Lewis et al., 2005; Ji et al., 2017).

The present study revealed that p62, WDFY3, NQO1, and LC3 were positive in the fatty area, ADM, and pancreatic cancer cells. As LC3 is incorporated into the inner and outer membrane of autophagosomes, detection of LC3-positive cells indicates only the presence or formation of autophagosomes (Kabeya et al., 2000). An increase in LC3 staining may indicate autophagy suppression; however, autophagic activity cannot be predicted based solely on the staining intensity of LC3. Therefore, evaluating autophagy activity or dysfunction based solely on LC3 is not appropriate. However, we observed an accumulation of p62 in acinar cells in regions with fatty infiltration, which should be degraded as an autophagy substrate. Therefore, autophagy dysfunction may occur in pancreatic acinar cells in regions with fatty infiltration. It is suggested that in fatty pancreas, fatty infiltration of the pancreatic acinus induces autophagy dysfunction, resulting in overexpression of p62 and NQO1, an antioxidant protein. However, p62 accumulation cannot be ruled out as other causes such as proteasome dysfunction, abnormal protein synthesis, and oxidative stress (Tang et al., 2019; Sánchez-Martín et al., 2020).

Further examination is needed to clarify the molecular mechanism involving the increase in the expression of LC3 and p62 in the acinar cells in fatty pancreas. The present research had the limitation of being only a clinicopathological study using resected pancreas, with the details of the molecular mechanism of impaired autophagy in fatty pancreas being unknown. Further basic research is needed to elucidate the pathogenesis of fatty pancreas.

## 5 Conclusion

In NAFLD patients, age and hypertension were risk factors for fatty pancreas. The risk factors for fatty pancreas in IPMN patients were being male and exhibiting hypertension, while the common risk factor for both groups was hypertension. In fatty pancreas, fatty infiltration into the pancreatic acinus might induce autophagy dysfunction, resulting in overexpression of p62 and NQO1, an antioxidant protein. Given that fatty pancreas may be a risk factor for pancreatic cancer, patients with fatty pancreas require careful follow-up.

## Data Availability

The original contributions presented in the study are included in the article/supplementary material, further inquiries can be directed to the corresponding author.
